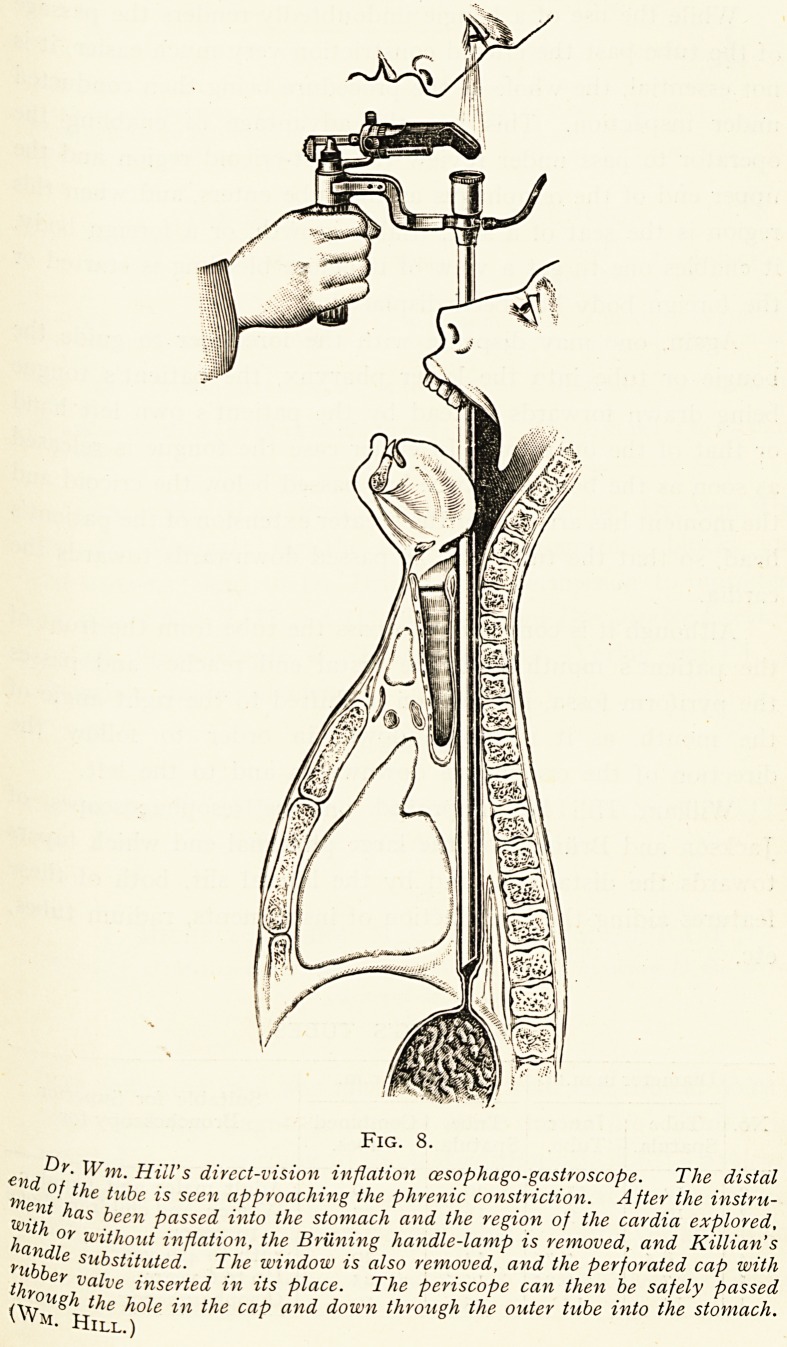# Direct Laryngoscopy, Bronchoscopy and Œsophagoscopy

**Published:** 1912-03

**Authors:** P. Watson-Williams

**Affiliations:** Lecturer on Otology, Rhinology, and Laryngology in the University of Bristol; and in Charge of the Ear, Nose and Throat Departments at the Bristol Royal Infirmary


### DIRECT LARYNGOSCOPY, BRONCHOSCOPY AND ŒSOPHAGOSCOPY.

BY

P. WATSON-WILLIAMS, M.D.Lond.,

*Lecturer on Otology, Rhinology, and Laryngology in the University of Bristol; and in Charge of the Ear, Nose and Throat Departments at the Bristol Royal Infirmary.*

---

BRONCHOSCOPY owes its inception to Killian of Freiberg and to Von Schrötter of Vienna, while other laryngologists—more particularly Chevalier Jackson and Brüning — have developed and modified the earlier instruments, both for the direct inspection and treatment of the larynx, bronchi and œsophagus. For the removal of foreign bodies from the trachea, bronchi and œsophagus, we now usually resort to direct inspection and removal through the mouth; while for diagnostic purposes, the direct inspection of neoplasms and the removal of fragments for histological examination, dilation of strictures, etc., bronchoscopy and œsophagoscopy have proved of the greatest value. Nothing illustrates more forcibly the far-reaching possibilities of these newer methods than the successful removal of a nail from the second division of a bronchus, and the introduction of a gold collar to overcome the cicatricial contraction that had arisen from the long-continued presence of the nail, a feat accomplished by Killian five years ago. The object of this article is to indicate very briefly the main points in the technique without entering on later developments, such as Brüning's tracheo- and broncho-graphy.

FIG. 1.
*Œsophagoscopic appearance of a malignant growth causing stricture 20 c.m. from the teeth.* (AUTHOR'S CASE.)

### Direct Laryngoscopy, Bronchoscopy and Œsophagoscopy.

*Direct Laryngoscopy.* — Kirstein in 1894 introduced the method of inspecting the larynx without the intervention of a mirror to reflect the light thrown into the back of the throat, so that the inspector's eye could look directly at the larynx instead of at its inverted image in a laryngoscopic mirror, the Kirstein method being termed direct laryngoscopy in contradistinction to indirect laryngoscopy.

*Light.*—Instead of using a concave forehead mirror, whereby the light is focussed on to the laryngoscopic mirror, Kirstein invented a lamp which reflects parallel rays from a small plane mirror attached to a forehead band. Kirstein's lamp is very convenient for use with short tubes, and is therefore often preferred for inspection and for operations on the larynx, a Brüning or a Schrötter lamp, or one with the light at the distal end, *e.g.* Chevalier Jackson's lamp, being reserved by some operators for the deeper lying trachea, bronchi or œsophagus.

Brüning has introduced a forehead lamp to replace Kirstein's. It is like a small Klar focus lamp, and gives a more powerful light than Kirstein's.

The fish-tailed tube spatula devised by Killian is perhaps the most convenient form for general use. One disadvantage in Killian's spatula lies in the fact that the handle being at right angles to the spatula it is not so easy to exert continuous traction on the distal end, so as to draw the hyoid bone and the structures at the base of the tongue forward without levering the instrument against the upper incisors. Hence Jackson introduced a double-rectangular tube spatula, with the handle parallel with the spatula, the form of handle adopted by Brüning.

*Technique.*—Before referring to the different methods of using these various direct laryngoscopic lamps, the question of local anæsthesia may be considered, for even when a patient is placed under general anæsthesia it is necessary to partially anæsthetise the fauces and larynx and, when the air passages are particularly irritable, to overcome retching and glottic spasm. It is well to follow Killian's recommendation to give a small dose of morphine, or in the case of a child codein, hypodermically about one hour before the operation.

FIG. 2.

DIAGRAM OF DIRECT LARYNGOSCOPY.

*The larger figure shows the laryngoscopic tube-spatula. The smaller figure, on the left, shows the tracheal tube-spatula in use, prior to its being passed between the vocal cords, after which the extension tube is passed through the lumen of the tube-spatula for entering a bronchial tube.* (WM. HILL.)

Direct laryngoscopy under local anæsthesia is generally possible in most adult patients. A 10 to 20 per cent. solution of cocaine hydrochlorate may be used, with or without a small percentage of suprarenal extract, which is advocated in order to prevent to some extent general absorption of the cocaine. I generally use 20 per cent. cocaine solution without any adrenalin, as it acts more quickly, and appears to be less likely to cause symptoms of cocaine poisoning than double the quantity of the weaker solution, and this has the advantage of avoiding the possibility of local trouble from the after effects of adrenalin.

FIG. 3.

*Direct Laryngoscopy for removal of a subglottic growth in a child under general anæsthesia.* (WM. HILL.)

A very small quantity is sprayed on to the fauces, and a few minutes later the cocaine is sprayed directly into the larynx by means of a fine laryngeal spray. As soon as the supraglottic

PLATE I.

BRONCHOSCOPY IN SITTING POSTURE OF PATIENT.

FIG. A. FIRST STAGE.

The patient holding his tongue out with the left hand, while

FIG. B. SECOND STAGE.

The tube spatula having been passed behind the portion of the larynx is anæsthetised more cocaine is applied by a laryngeal swab passed between the vocal cords. In three or four minutes the patient is then ready for direct inspection.

*Position of the patient and examiner.*—The patient's collar, and all tight garments, stays and so forth, having been loosened, inspection may be made with the patient sitting up or lying on a table. In the former case the patient sits on a low stool about twelve inches high, a nurse or assistant standing behind so that the patient's back is supported by the assistant's knees, while the patient's head is extended well back and supported in that position by the assistant's hands, as shown in the accompanying illustrations. The examiner stands in front of the patient, holding the lamp in the right hand, while the left is used to raise the upper lip or moustache should they be in danger of being caught between the tube and the upper incisor teeth.

When the patient is examined lying down, under local anæsthesia, the right or left lateral position is chosen.

It is well to come to a definite understanding with the patients that, if for any reason they feel they cannot tolerate the continuance of the inspection, the instrument will be withdrawn at a given signal, and on no account should such a pre-arranged signal be disregarded. While this is the more essential in bronchoscopy and œsophagoscopy, when it is obvious that only by the absolute confidence of the patient being maintained is it possible to safely pass a long stiff metal tube into the trachea, bronchus or œsophagus, it is also helpful in the simpler procedure of laryngoscopy. If the patient is absolutely convinced that at any moment he gives the signal the instrument will be withdrawn, he has courage to tolerate their introduction as long as possible.

With the lamp lighted, the tube spatula should be guided nearly horizontally to the posterior pharyngeal wall, and then directed vertically down behind the epiglottis, which the end of the spatula is made to press forwards till the arytenoid eminences and then the vocal cords are brought into view, so that the whole of the larynx and the infra-glottic trachea come under inspection.

The tube spatula for an adult should be about 15 to 20 c.m. in length (6 to 8 inches), but for children one about 15 c.m. (6 inches) long, or somewhat less, is sufficient; the laryngeal end, however, is narrower for children, as the small larynx will not allow of the introduction of the wider tube suitable for adults. But owing to the small size of the larynx in very young children, the narrowness of the glottic chink, and the delicacy of the mucous membrane, it is often convenient and sometimes safer to use a laryngeal tube spatula with a bifid extremity, which is placed in the glosso-epiglottic fossæ, so that the base of the tongue being drawn forwards the epiglottis is raised and drawn forwards at the same time, so as to give a good direct view of the larynx.

Fig. 4.

*Scheme showing average measurements in adult males from the upper molar teeth to the bifurcation of the trachea, etc.* (after Waggett).

In order to overcome the difficulty of operating through a very narrow tube I have devised one with a distal end narrow enough to allow of its introduction into the larynx of children from five years onwards, but with a lumen of one inch wide at the proximal end, the lumen tapering to laryngeal end. This not only facilitates the introduction of instruments at the side of the lumen, so as not to obstruct the view of the operation field, a point of great importance in the case of very young children, but has further the great advantage of allowing binocular vision.

PLATE II.

TRACHEO-BRONCHOSCOPY IN THE DORSAL POSTURE.

FIG. A. STAGE I.

*Note the oblique direction of the instrument when being passed behind the epiglottis.* (BRÜNING.)

FIG. B. STAGE II.

*The tube spatula having entered the larynx, the patient leaves hold of the tongue, while the tube is passed horizontally through the glottis into the trachea* [illegible] (BRÜNING.)

Fig. A.

*Position for exploring and operating under chloroform on the larynx and air passages. In œsophagoscopy and gastroscopy the patient's head must be held by an assistant to facilitate raising and lowering as the end of the tube advances through the various curves of the gullet.* (Wm. Hill).

Fig. B.

*Tracheo-bronchoscopy in the left lateral posture.* (Brüning.)

General anæsthesia is seldom required for diagnostic purposes in the adult, but is nearly always necessary in the case of children. But with nervous patients it is usually necessary to resort to general anæsthesia for operations on the larynx by the direct method.

For direct laryngoscopy under general anæsthesia the patient may be in the dorsal, right lateral, or left lateral position. In a child the head is more readily extended, and with the head hanging over the end of the table the dorsal position is usually most satisfactory; but the lateral position is generally to be preferred in adult patients; whether the patient is made to lie on the right or left side is largely a matter of custom. In Germany the right lateral position is usually preferred, but I find the left lateral position is the more convenient, as it appears to me easier to guide the laryngeal spatula with the left hand, with the patient in this position, leaving the right free for manipulations, whereas in the right lateral position, either the left hand must be passed over the patient's face, so as to grasp the handle of the lamp with the back of the hand uppermost, or else the spatula is passed with the right hand, and then, when the larynx is exposed, the hands are changed so as to set free the right hand for manipulations.

*Direct Tracheoscopy and Bronchoscopy.*—*In superior bronchoscopy* the tube is introduced through the glottis, and thence into the right or left bronchus, and even in rarer conditions into the second divisions, whereas in *lower bronchoscopy,* the tube is introduced through a tracheotomy wound. In the former case the diameter of the tube is restricted by the dimensions of the glottis, and the tube has a longer path to traverse before it reaches the end of the trachea than in lower bronchoscopy, which has the advantage of allowing a tube of greater diameter. Obviously the diameter and length of the tube that can be employed depends on the age of the patient, whether a preliminary operation has been performed or not, and on the situation of the foreign body or lesion.

| | Average Distances from* | | | | | |
|---|---|---|---|---|---|---|
| | Incisors to Vocal Cords. | Vocal Cords to Bifur-cation. | Diam. of Tube. | Diam. of Trachea. | Diam. of R. Bron. | Diam. of L. Bron. |
| Adult Man .. .. | 11 c.m. or $4\frac{1}{2}$ in. | 11 c.m. or $4\frac{1}{2}$ in. | 9 to 11 m.m. | 16 to 23 m.m. | 15 to 22 | 20 |
| Child, 10 years .. | .. | .. | .. | 10 m.m. or in. | .. | .. |
| Child, 5 years .. .. | .. | .. | .. | 5 m.m. or $\frac{1}{5}$ in. | .. | .. |

* From data cited by Waggett, Allbutt's *System*, vol. iv.

For *superior bronchoscopy* a tube spatula with a bevelled end is introduced under inspection through the glottis. The patient slightly retracts the head and holds the tongue out with the left hand, until the instrument has been passed over the epiglottis. Then the patient lets his tongue go, and retracts his head as far as he can while the spatula is directed in a more vertical direction downwards through the glottis. Of course, under general narcosis the holding of the tongue and extension of the head is carried out by an assistant.

With the Brüning instrument for adults, use a tube about 18 to 22 c.m. long and 9 m.m. in diameter ; for children about 13 to 18 c.m. long and 7 m.m. or less in diameter ; then, having further cocainised the lower trachea, the corresponding extension tube is passed down inside the tube spatula as far as may be necessary, until the foreign body, etc., is reached, either in the trachea, right or left bronchus, as the case may be. Swabbings may be required to sponge out the mucus. This is readily done by passing the previously prepared cotton swabs on the long holders. Needless to say, great care must be observed to ascertain that the swab is firmly fixed before it is introduced.

The carina, or division between the two main bronchi, is to the left of the tracheal axis, and the right bronchus diverges so much less than the left so as to seem to be more or less a continuation of the trachea, while the left bronchus diverges at a greater

PLATE IV.

FIG. A.

*Direct tracheoscopy in dorsal position, Killian's tube spatula.* (KILLIAN.)

FIG. B.

*Direct tracheoscopy with Killian's tube, sitting posture.* (KILLIAN.)

angle from the perpendicular. Hence foreign bodies are much more prone to find a ready entrance into the right bronchus than the left.

These facts should be borne in mind when one has to introduce the bronchoscopic tube into the right or left bronchus. Hence if the patient's head be tilted to the right as soon as the tube has passed down the trachea to near the lower end, while at the same time the proximal end is brought well over to the right of the middle line, causing the distal end to draw the lower end of the trachea towards the left, the tube as it passes obliquely down is made to enter the left bronchus. There is seldom much difficulty in passing the tube into the right bronchus, but in order to avoid injuring the carina one reverses the direction of the instrument, making the head and upper end tilt to the left so as to direct the lower end towards the right bronchus.

*Lower Bronchoscopy*, or bronchoscopy through a tracheotomy wound, is much simpler and easier than superior bronchoscopy, but involves a previous tracheotomy. Occasionally this can be done at a sufficient interval before the examination with the bronchoscope to enable the wound to heal, but more usually the passage of the bronchoscopic tube is for the immediate removal of a foreign body which cannot be extracted *per vias naturales*. Under these circumstances it is important to cause as little bleeding as possible, hence Waggett urges the desirability of the bloodless method; that is to say, after the incision of the skin the operation is completed by a pair of blunt hooks, retracting the soft tissues until the trachea is exposed and incised, the thyroid isthmus, if necessary, being divided and ligatured.

*Œsophagoscopy.*—Before introducing the œsophagoscope the lower end of the pharynx should have been inspected by an ordinary laryngoscopic mirror, and in addition such information as may be gathered from von Eicken's method of hypo-pharyngoscopy is always desirable, so that the existence of any ulceration or growth in the post-cricoid region is already known. Moreover, the patient should have been carefully examined for any indication of aortic aneurysm, mediastinal tumour, or other lesion which might add to the risk of œsophagoscopy. To obviate trouble from the regurgitation of food during the examination, the patient should have fasted for at least four hours, and it is an advantage, especially with nervous patients, to have injected a small dose of morphine about an hour before the examination, as recommended by Killian for bronchoscopy.

FIG. 5.

*Diagram showing position of the laryngoscopic mirror in viewing the hypopharynx, which is exposed to view by the forward traction exerted by means of Von Eicken's probe inserted between the glottis so as to draw forwards the hyoid bone.*

The patient may be in the sitting position, or lying on a table in the right or left lateral position, and, as in bronchoscopy, the patient's collar, and of course any tooth plate, must be removed, and all tight clothing about the neck loosened. In the sitting position the same low stool is required, and the patient is supported by an assistant from behind. If the patient is on his side, lying on a table, the head should project over the end, supported by an assistant, so that it can be elevated or lowered as the tube is passed down the varying curves of the œsophagus.

The œsophagus in the adult male has a diameter of approximately one inch, but except at the extremities it is very distensile. At the upper end, behind the lower border of the cricoid cartilage, it is a transverse slit, and its cardiac end is a slit lying obliquely from behind, forwards, and to the left. Its long axis passes slightly to the left, from above downwards, the average distances from the teeth to the cricoid and thence to the cardia being given in the table appended.

The œsophagus is fairly tolerant of the passage of the tube, but the fauces and lower pharynx may be rendered insensitive by cocaine, 10 per cent. solution sprayed sparingly, a small quantity being swallowed by the patient making the introduction of the tube down the œsophagus much more readily borne.

For an adult one should begin with a short tube, say 26 m.m. long by 13 m.m. diameter, warmed and oiled, and having a closely-fitting, soft, round-ended bougie, introduced so that it projects for an inch or two through the lower end of the lumen.

In the case of a patient sitting on a stool, the operator faces the patient, holding the lamp and tube in his right hand; the left forefinger may be introduced into the patient's right pyriform fossa, the tube with the projecting bougie being passed backwards through the right angle of the mouth, and guided by the finger-tip backwards into the pharynx.

Up to this point the patient's head is very slightly extended, thus differing from the position in bronchoscopy; but as soon as the bougie passing behind the cricoid cartilage enters the œsophagus the left forefinger is withdrawn, the patient is told to swallow, and the head is more extended as the tube is gently passed down and the bougie withdrawn. Thereafter the downward passage of the tube is guided by inspection, careful note being made of any abnormality in the œsophagal walls, and as the tube is gradually withdrawn the walls of the œsophagus are again subject to inspection. If no obstruction has been encountered, and the seat of lesion is farther down, a second tube of greater length is re-introduced down to and through the cardia, any mucus or gastric contents which regurgitate into the lumen being removed by swabbing or by means of the suction pump.

FIG. 6.

*Diagram (after Waldeyer-Joessel) to show the relation of the œsophagus to the trachea left bronchus and aorta, and especially* [illegible]

FIG. 7.

*Diagram (after Corning) to show the various curves and constrictions of the œsophagus. Where the lowest horizontal line meets the dotted line marks the level of the hiatus œsophagus of the diaphragm ; below this the subphrenic gullet deviates to the left. This deviation must be borne in mind in passing the outer œsophago-gastric tube through the lower part of the gullet into the* [illegible]

FIG. 8.

*Dr. Wm. Hill's direct-vision inflation œsophago-gastroscope. The distal end of the tube is seen approaching the phrenic constriction. After the instrument has been passed into the stomach and the region of the cardia explored, with or without inflation, the Brüning handle-lamp is removed, and Killian's handle substituted. The window is also removed, and the perforated cap with rubber valve inserted in its place. The periscope can then be safely passed through the hole in the cap and down through the outer tube into the stomach.* (WM. HILL.)

While the use of a bougie undoubtedly renders the passage of the tube past the cricoid constriction very much easier, it is not essential, the whole of the procedure being then conducted under inspection. This has the advantage of enabling the operator to pass under review the post-cricoid region and the upper end of the œsophagus as the tube enters, and when this region is the seat of a soft, friable growth, or a foreign body, it enables one to get a view of it before bleeding is started or the foreign body has been displaced.

Again, one may dispense with the forefinger to guide the bougie or tube into the lower pharynx, the patient's tongue being drawn forwards instead by the patient's own left hand or that of the operator. In either case the tongue is released as soon as the bougie or tube has passed below the cricoid and the moment has arrived for the greater extension of the patient's head, so that the tube may be passed downwards towards the cardia.

Although it is convenient to pass the tube from the front of the patient's mouth until the distal end reaches and passes the pyriform fossa, its position is shifted to the right angle of the mouth, as it is passed down, in order to follow the direction of the œsophagus downwards and to the left.

William Hill has improved on the œsophagoscopes of Jackson and Brüning by the large proximal end which tapers towards the distal end, and by the lateral slit, both of these features aiding the introduction of instruments, radium tubes, etc.

BRÜNING'S TUBES.

| No. | Diameter in m.m. | | Length in c.m. | | Suitable for Superior Bronchoscopy for |
|---|---|---|---|---|---|
| | Tube Spatula. | Inner Tube. | Tube Spatula. | Combined Tubes. | |
| 2 | 12 | 11 | 20 | 40 | Adult males and females. |
| 3 | 10 | 9 | 18 | 37 | Women, and children above 10 years. |
| 4 | 8.5 | 7.75 | 14 | 30 | 12th to 4th year. |
| 5 | 7 | 6.25 | 11.5 | 23 | Up to 6 years. |
| 1 | 14 | 13 | 25 | 50 | Œsophagoscopy. |

TABLE OF DISTANCES (BRÜNING).

| | Men. | Women. | Child (aged 10) |
|---|---|---|---|
| From upper incisors to glottis .. .. .. .. | 14 c.m. | 13 c.m. | 10 c.m. |
| From glottis to bifurcation .. .. .. .. | 12 c.m. | 10 c.m. | 7 c.m. |
| From upper teeth to bifurcation .. .. .. | 26 c.m. | 23 c.m. | 17 c.m. |

TABLE OF DIAMETERS (BRÜNING).

| | Men. | Women. | Child (aged 10). | Infant. |
|---|---|---|---|---|
| Trachea .. .. | 15 to 22 m.m. | 13 to 18 m.m. | 8 to 11 m.m. | 6 to 7 m.m. |
| Right main bronchus. | 12 to 16 m.m. | 10 to 15 m.m. | 7 to 9 m.m. | 5 to 6 m.m. |
| Left main bronchus. | 10 to 14 m.m. | 9 to 13 m.m. | 6 to 8 m.m. | 4 to 5 m.m. |
| Extreme width of glottis. | 12 to 15 m.m. | 10 to 13 m.m. | 8 to 10 m.m. | 5 to 6.5 m.m. |

I am indebted to Prof. Killian, Dr. Brüning, Dr. Wm. Hill and Dr. Waggett for the loan of photographs, blocks and illustrations; and to Dr. Brüning for permission to reproduce the plates Nos. I, II and III (Fig. B) from *Die Direkte Laryngoskopie, Bronkoskopie u. Œsophagoskopie* (Verlag von J. F. Bergmann in Wiesbaden).

## Figures and Tables

**Fig. 1. f1:**
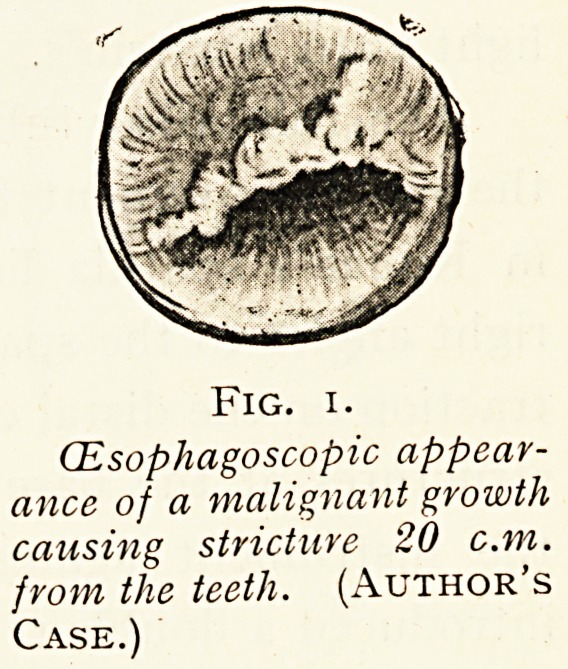


**Fig. 2. f2:**
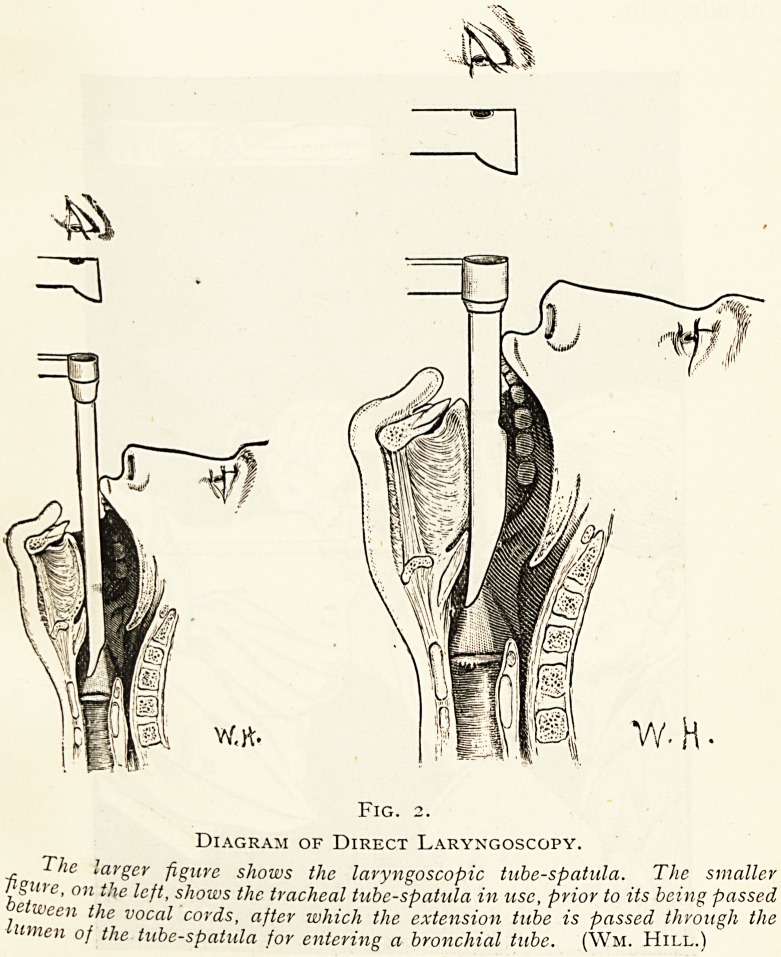


**Fig. 3. f3:**
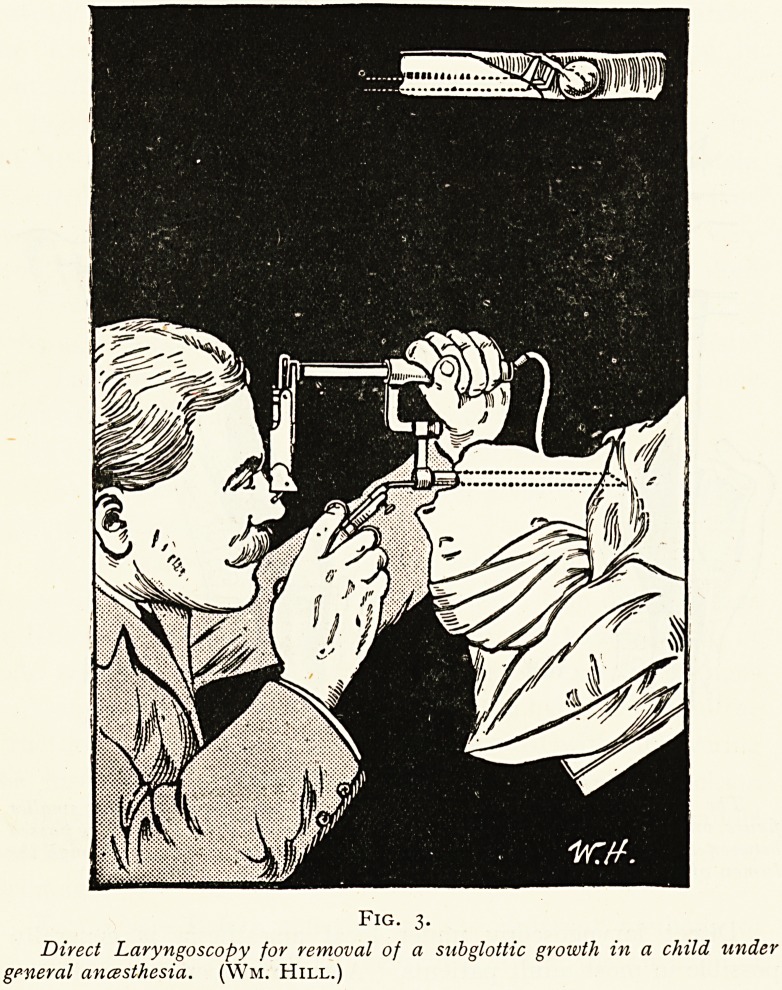


**Fig. A. Fig. B. f4:**
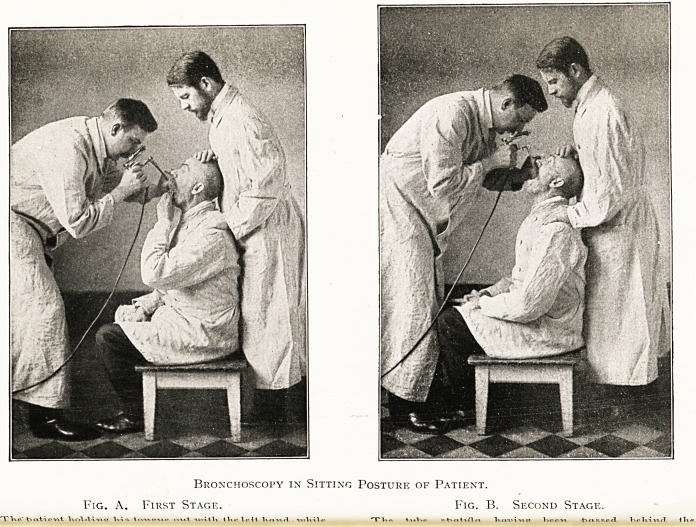


**Fig. 4. f5:**
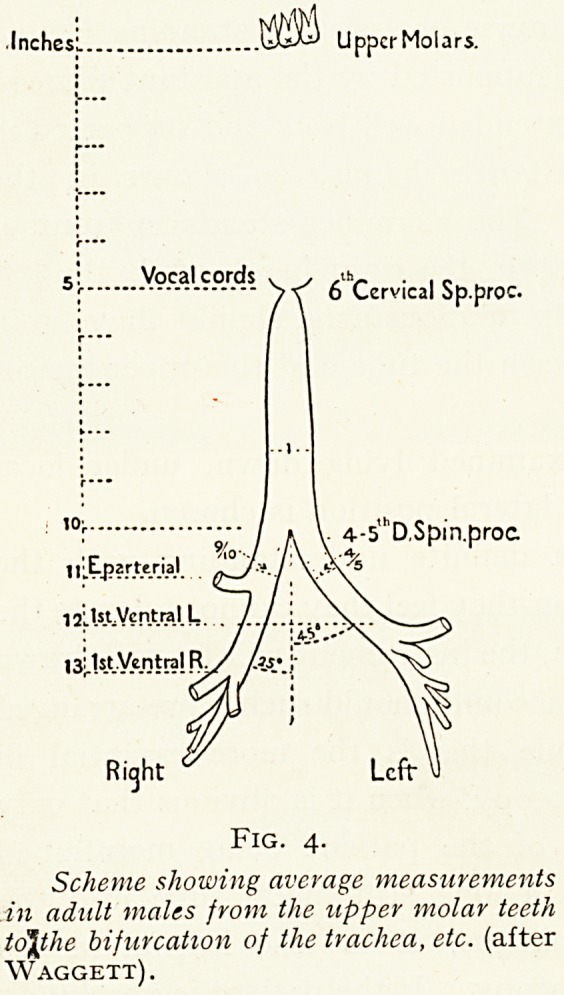


**Fig. A. Fig. B. f6:**
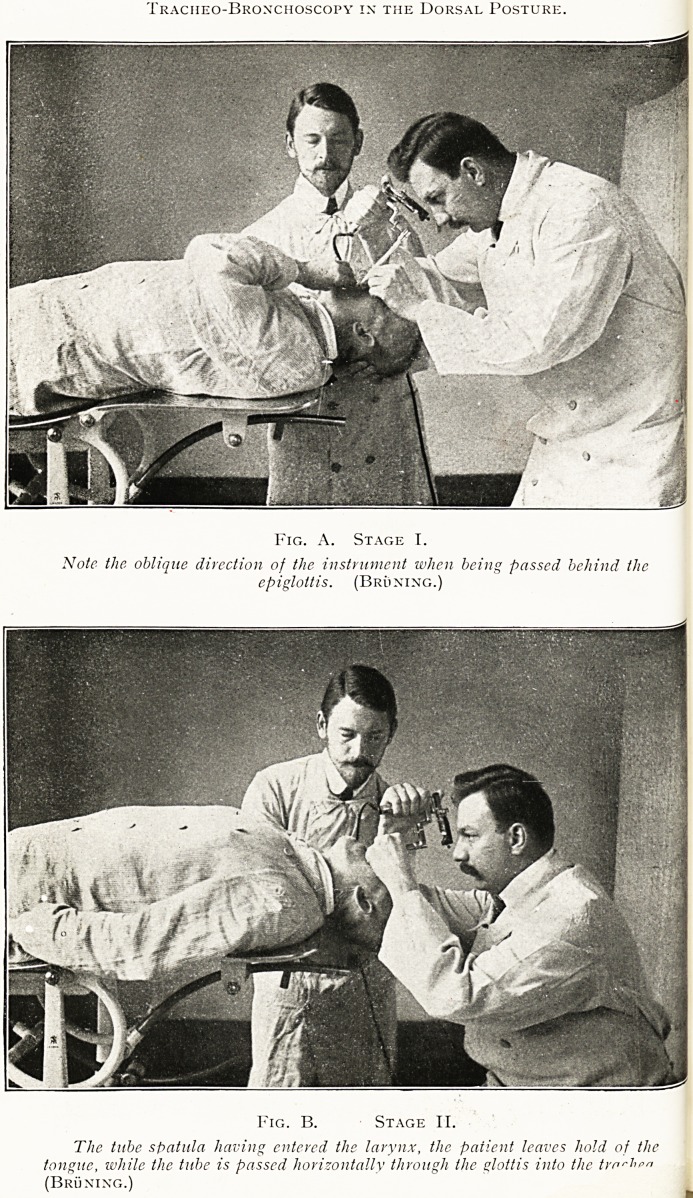


**Fig. A. f7:**
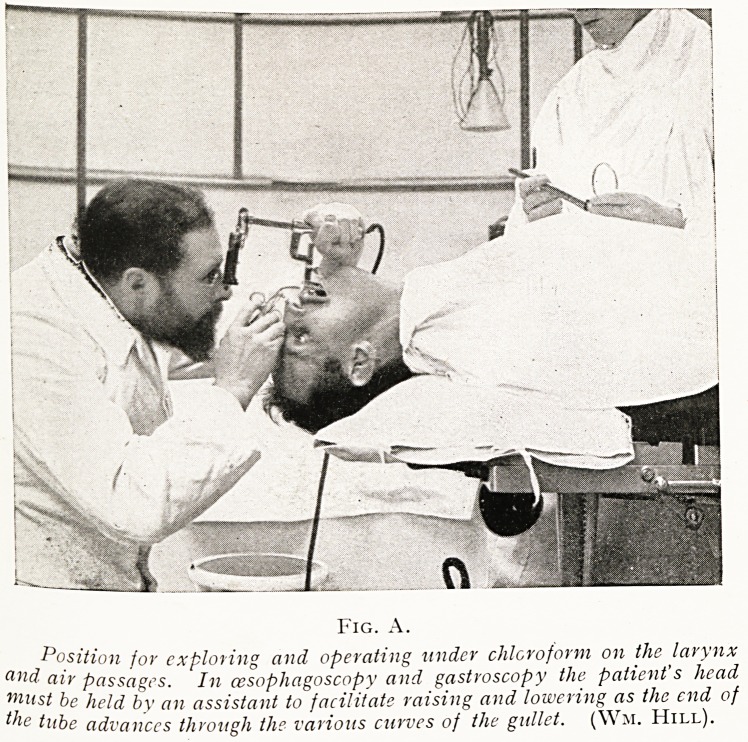


**Fig. B. f8:**
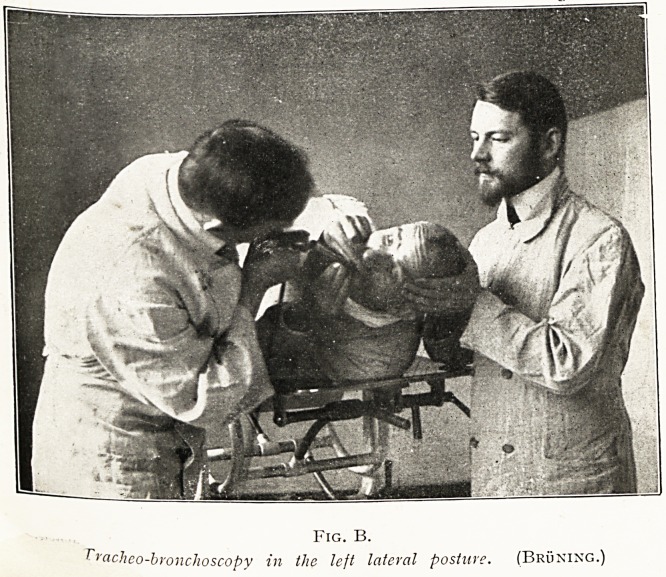


**Fig. A. f9:**
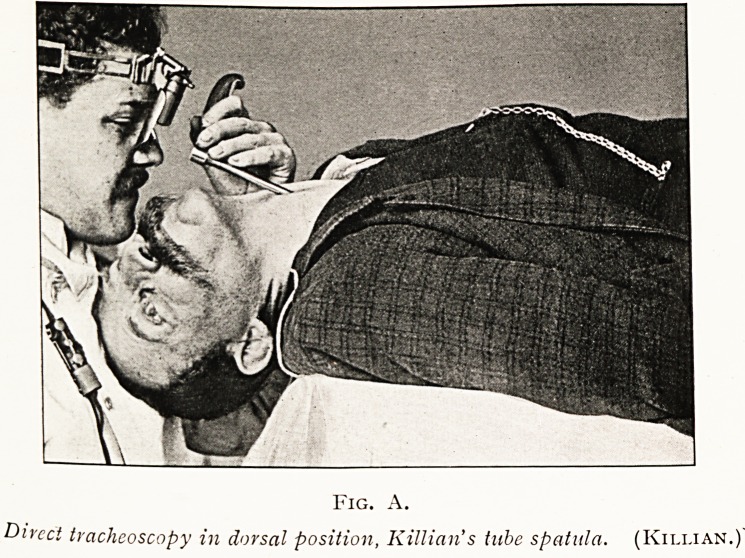


**Fig. B. f10:**
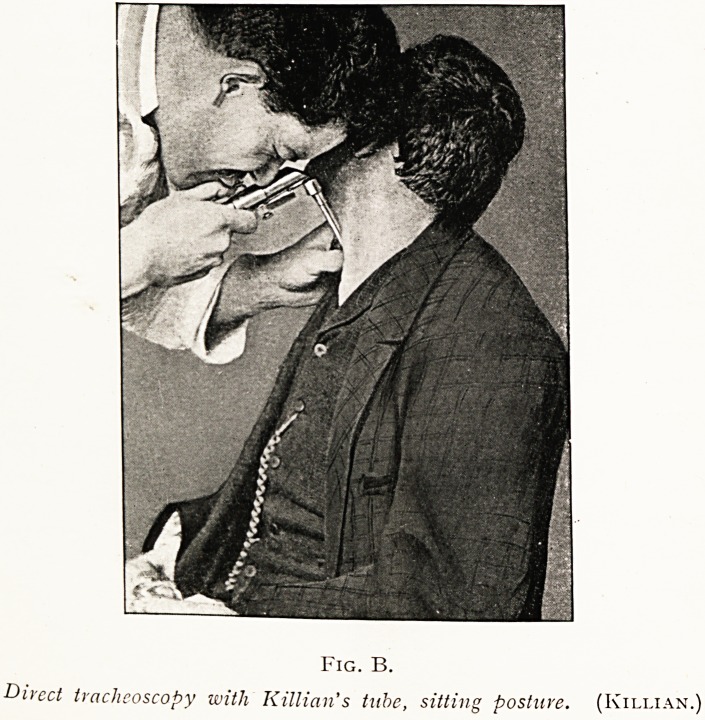


**Fig. 5. f11:**
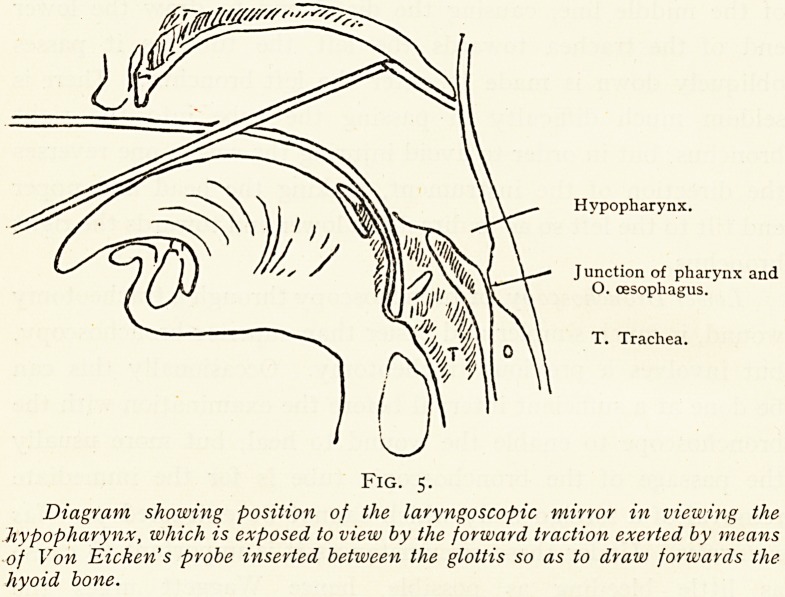


**Fig. 6. f12:**
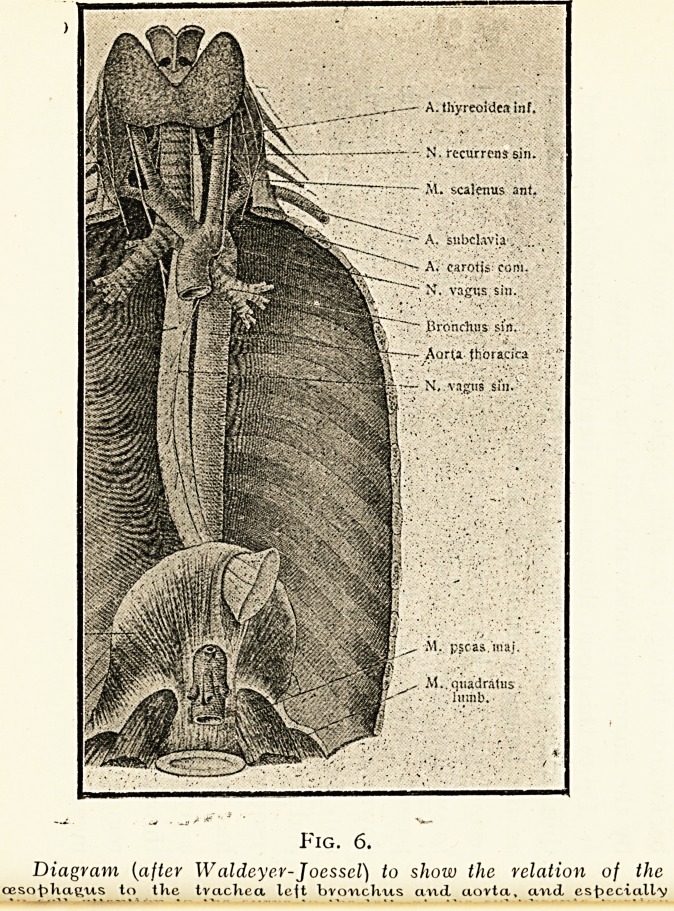


**Fig. 7. f13:**
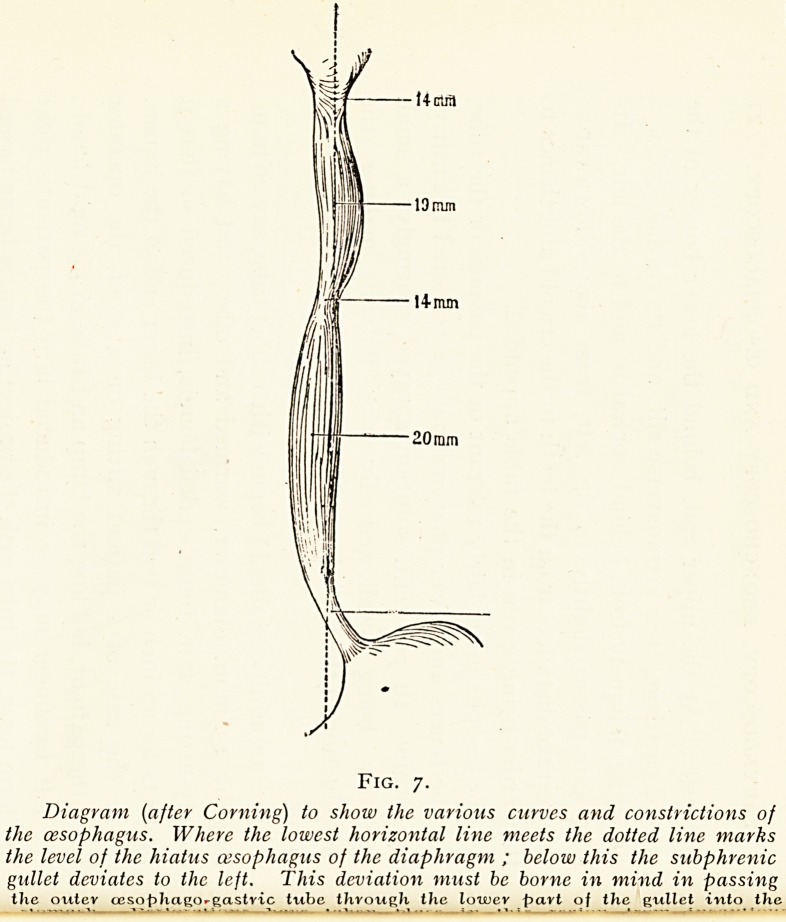


**Fig. 8. f14:**